# Environmental DNA Insights into the Spatial Status of Fish Diversity in the Mainstem of the Jialing River

**DOI:** 10.3390/ani15010105

**Published:** 2025-01-05

**Authors:** Xinxin Zhou, Xiaohan Dong, Jiaxin Huang, Shuli Zhu, Weitao Chen, Yanjun Shen

**Affiliations:** 1 Laboratory of Water Ecological Health and Environmental Safety, School of Life Sciences, Chongqing Normal University, Chongqing 401331, China; zhouxinixin726@163.com (X.Z.); 18325131947@163.com (X.D.); 19971464829@163.com (J.H.); 2 Chongqing Key Laboratory of Conservation and Utilization of Freshwater Fishes, School of Life Sciences, Chongqing Normal University, Chongqing 401331, China; 3 Pearl River Fisheries Research Institute, Chinese Academy of Fishery Sciences, Guangzhou 510380, China; zshuli2009@126.com

**Keywords:** environmental DNA, anthropogenic activities, fish diversity, community composition

## Abstract

Preserving healthy river habitats is essential for maintaining fish diversity. Over time, anthropogenic activities have severely damaged river habitats, leading to notable changes in fish diversity patterns. Conducting thorough and reliable investigations into fish diversity is crucial for assessing anthropogenic impacts on diversity. In August 2023, a water ecology survey was conducted across 20 terraced river sections in the mainstem of the Jialing River, resulting in the collection of 60 environmental DNA water samples. The survey identified 99 fish species, representing 74 genera across 7 orders and 20 families, with the Cyprinidae exhibiting the highest number of species. The fish communities are predominantly composed of species inhabiting slow flowing water, demersal fish, omnivores, and fish spawning adhesive eggs. Overall, small-bodied fish dominate the mainstem of the Jialing River, and the species preferring flowing habitats are relatively scarce. In addition, geographic division analyses revealed minimal variations in fish species composition and diversity among the terraced reservoirs and across the upper, middle, and lower reaches. Notably, the fish compositions in the middle and lower reaches were found to be similar, indicating a certain degree of convergence in these sections of the Jialing River. In conclusion, this study unveils the current status and distribution pattern of fish diversity in the Jialing River and highlights the extent of anthropogenic activities’ impact on fish diversity.

## Simple Summary

Biodiversity serves as a crucial safeguard for the stability of ecosystems. Currently, the significant decline in biodiversity stands as one of the most pressing challenges we encounter. Fish communities are a vital component of biodiversity, and protecting fish diversity contributes to the conservation of overall biodiversity. Therefore, this study utilizes environmental DNA technology to explore the fish diversity in the mainstem of the Jialing River. The findings illuminate the current status of fish diversity in the Jialing River and highlight the impact of human activities on it.

## 1. Introduction

Biodiversity plays a crucial role in maintaining ecosystem stability and regulating essential ecosystem functions [1,2]. Since the 20th century, one of the most pressing global challenges has been the significant decline in biodiversity [3]. Fish communities are among the most diverse within aquatic ecosystems [4], intricately connected to their surrounding aquatic habitats and serving as vital components of biodiversity [5]. Fish are an irreplaceable part of aquatic ecosystems, and protecting their diversity is fundamental to preserving ecosystem stability and health [6,7].

River connectivity is a critical factor influencing species distribution and survival [8]. Naturally flowing rivers offer a diverse array of heterogeneous habitats for organisms and are critical for the functioning of river ecosystems and the preservation of biodiversity [9,10]. However, human activities, such as dam construction [11], overfishing [12], water pollution [13], and deforestation along riverbanks [14], have long impacted natural river systems, thereby affecting fish diversity. Among these activities, dam construction has emerged as a significant driver of ecosystem alteration [11]. In China, the proliferation of terraced hydropower projects has fragmented numerous rivers with multiple dams [15]. This fragmentation disrupts river continuity, resulting in habitat modifications and transforming continuous, heterogeneous flowing water environments into homogenized, semi-lentic habitats [16]. Changes in the hydrological environment, particularly those affecting fish species that spawn drifting eggs, can hinder embryo development due to inadequate drift time in semi-lentic waters [17]. Furthermore, dams obstruct fish migration routes [18], leading to population segregation and reduced gene flow factors that have profound implications for fish populations [19]. Additionally, dams also prevent juvenile fish from the upper reaches from migrating to the foraging grounds in the lower reaches, severely affecting their growth and development and potentially leading to the miniaturization of certain fish species [20]. Consequently, river fragmentation undermines the capacity of fish populations to adapt to environmental changes and disrupts community structure stability, posing a significant threat to fish diversity [21].

Historically, extensive surveys of riverine fish diversity and communities primarily relied on conventional techniques such as electrofishing, gillnetting, and trawling [22,23]. However, these traditional methods are not only costly and time-consuming but also highly invasive, potentially disrupting aquatic ecosystems [24,25,26]. Moreover, their effectiveness can be influenced by various factors, including weather conditions, fish size, and behavior [27,28]. In contrast, environmental DNA (eDNA) metabarcoding technology has shown significant advantages in monitoring species and community distribution in natural rivers, estimating species abundance, and assessing biodiversity [29,30,31]. This technology is non-invasive, highly sensitive, and cost-effective, effectively addressing many of the limitations associated with traditional methods [32]. Furthermore, studies have demonstrated that the results obtained from eDNA monitoring closely align with those derived from traditional survey methods [33,34].

**Figure 1:**
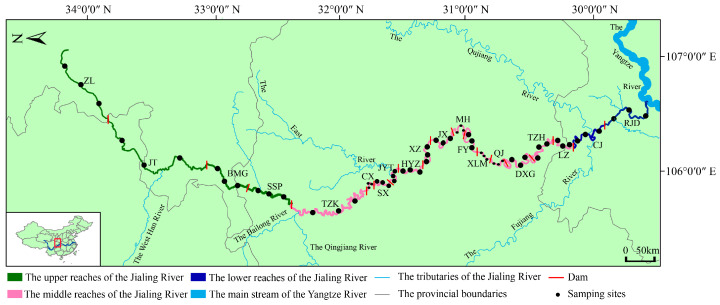
Sampling sections of the mainstem of the Jialing River (ZL: Zhu Lin; JT: Ju Ting; BMG: Ba Miaogou; SSP: Shang Shipan; TZK: Ting Zikou; CX: Cang Xi; SX: Sha Xi; JYT: Jin Yintai; HYZ: Hong Yanzi; XZ: Xin Zheng; JX: Jin Xi; MH: Ma Hui; FY: Feng Yi; XLM: Xiao Longmen; QJ: Qing Ju; DXG: Dong Xiguan; TZH: Tong Zihao; LZ: Li Ze; CJ: Cao Jie; and RJD: Ru Jiangduan). The red square indicates the study area. Due to the shorter length of certain river sections, the size of the points has been appropriately adjusted. The varying sizes of the points do not carry any additional significance. The map of China data was obtained from http://bzdt.ch.mnr.gov.cn/ (accessed on 1 September 2023), and the map was created using ArcMap 10.8.

The Jialing River, the largest tributary in terms of basin area in the upper reaches of the Yangtze River, originates from the southern base of the Qinling Mountains in Feng County, Shanxi Province. It flows through Shanxi, Gansu, Sichuan, and Chongqing, stretching a total length of 1120 km and encompassing a basin area of approximately 160,000 km^2^ before merging into the Yangtze River at Chao Tianmen in Chongqing (Figure 1) [35]. However, anthropogenic disturbances along the watershed have significantly altered the original habitat characteristics of the Jialing River basin over time. Since the establishment of the Ma Hui Hydropower dam in 1970, numerous hydropower stations have been constructed along the Jialing River. Currently, there are 19 terraced hydropower dams on the mainstem of the Jialing River, with over 10 additional dams either under construction or in the planning phases. These dams not only block fish migratory routes but also profoundly alter the original aquatic ecological conditions of the Jialing River [35].

Currently, research efforts in the Jialing River basin primarily focus on conducting fish resource surveys in the middle and lower reaches of the river [36,37], often neglecting comprehensive studies on the overall status of the basin. As a result, these studies do not adequately reflect the impacts of anthropogenic activities on fish diversity in the mainstem of the Jialing River. To address this gap, the present study aimed to investigate the spatial status of fish diversity in the Jialing River mainstem in the context of anthropogenic activities. In August 2023, we monitored and analyzed fish species composition across 20 river sections using eDNA metabarcoding technology. This endeavor seeks to establish a scientific foundation for protecting fish diversity and maintaining fish resources in the Jialing River, while also providing essential data to understand the impact of anthropogenic activities on fish diversity.

## 2. Materials and Methods

### 2.1. Study Area

The mainstem of the Jialing River extends from 29°40′ N to 34°30′ N and from 105°70′ E to 107° E, featuring a natural descent of 2300 m. Its upper reaches are located upstream of Zhaohua District, Guangyuan City, Sichuan Province, while the middle reaches stretch from Zhaohua District to Hechuan District, Chongqing City. The lower reaches then extend from Hechuan to Chao Tianmen. For this study, we established 20 distinct river sections, each demarcated by 1 of the 19 terrace hydropower dams along the mainstem of the Jialing River. These sections serve as sampling areas and include the following 19 terraced reservoirs: Zhu Lin (ZL), Ju Ting (JT), Ba Miaogou (BMG), Shang Shipan (SSP), Ting Zikou (TZK), Cang Xi (CX), Sha Xi (SX), Jin Yintai (JYT), Hong Yanzi (HYZ), Xin Zheng (XZ), Jin Xi (JX), Ma Hui (MH), Feng Yi (FY), Xiao Longmen (XLM), Qing Ju (QJ), Dong Xiguan (DXG), Tong Zihao (TZH), Li Ze (LZ), Cao Jie (CJ), and Ru Jiangduan (RJD) (Figure 1). Geographically, ZL, JT, BMG, and SSP are situated in the upper reaches (UR) of the Jialing River, while CJ and RJD are in the lower reaches (LR), with the remaining reservoirs located in the middle reaches (MR). To obtain a more comprehensive and representative understanding of aquatic life across these 20 river sections, we designated three sampling sites within each area. These sites are positioned at the head, middle, and tail of each reservoir. The head site represents a deep, still water habitat, the middle site corresponds to a deep, slow-moving water habitat, and the tail site reflects a shallow, fast-moving water habitat.

### 2.2. eDNA Sampling and Enrichment

During the field sampling process, equal volumes of water samples were collected from the upper, middle, and lower water layers at each sampling site using a plexiglass water sampler, and these samples were then combined. The mixed samples were stored in sterilized polyethylene bottles and promptly refrigerated. To minimize the risk of contamination, all instruments were disinfected with a 10% bleach solution prior to collecting water samples from each sampling area [38]. Following disinfection, the instruments were rinsed with distilled water, washed with river water, and replaced with disposable gloves. In cases where sediment was present in the water samples, sterile medical gauze was used for pre-filtration during collection [39]. The pooled water samples from the three sampling sites within the same area were then further mixed and divided equally into three technical replicates. For each replicate, 2 L of water was filtered. Prior to filtering water samples in each sampling area, the filtering instruments were sterilized with a bleach solution to prevent potential cross-contamination. Three parallel water samples were simultaneously filtered through a 0.45 µm mixed cellulose filter membrane (Whatman, Maidstone, UK) within a 24 h period using a vacuum pump apparatus (SHZ-D(III), Shanghai, China). Distilled water served as a control for each river section to monitor for potential foreign DNA contamination. Finally, the filter membranes intended for subsequent DNA extraction were cryopreserved at −80 °C.

### 2.3. eDNA Processing and Sequencing

The procedure adhered to the instructions provided in the PowerWater DNA Isolation Kits (Omega, Norcross, USA) for extracting total DNA from the filter membrane. Each of the three samples was extracted separately, with a blank filter membrane serving as a negative control. The extracted DNA solutions were stored at −20 °C for subsequent PCR amplification.

PCR amplification was carried out using universal primers targeting the 12S rRNA locus of fish mitochondrial genes (Tele02-F: 5′-AAA CTC GTG CCA GCC ACC-3′; Tele02-R: 5′-GGG TAT CTA ATC CCA GTT TG-3′) [33,40,41]. Barcode sequences were added to the DNA of each sample to uniquely identify and differentiate DNA molecules from different samples. TransStart FastPfu DNA Polymerase (TransGen Biotech, Beijing, China) was used for the amplification process, with the following reaction system: 4 µL 5 × FastPfu Buffer, 2 µL (2.5 mmol∙L^–1^) dNTPs, 0.4 µL (2.5 units) FastPfu Polymerase (TransGen Biotech, Beijing, China), 1 µL template DNA (10 ng∙µL^–1^), and 0.8 µL (10 µmol∙L^–1^) each of forward and reverse primers. Finally, the volume of the mixture was adjusted to 20 µL using ddH_2_O. The PCR conditions were as follows: pre-denaturation at 95 °C for 5 min, denaturation at 95 °C for 30 s, annealing at 55 °C for 30 s, elongation at 72 °C for 45 s, final elongation at 72 °C for 10 min, and final storage at 10 °C (denaturation–annealing–elongation for 35 cycles). Additionally, a PCR negative control was included using ddH_2_O as a template for potential contamination during the amplification process. Each sample underwent three rounds of PCR amplification, and the resulting products were combined and subsequently analyzed via 2% agarose gel electrophoresis. In total, 60 samples were subjected to electrophoresis to identify the target band, which was approximately 167 bp in length, while none of the negative controls exhibited the target band. The PCR products were retrieved from gels using the Axyprep DNA Gel Recovery Kit (AXYGEN, Hangzhou, China) and then forwarded to Shanghai Lingen Biotechnology Co. in China for high-throughput sequencing using the Illumina NovaSeq 6000 sequencing platform (San Diego, CA, USA).

### 2.4. Bioinformatic Analyses and Taxonomic Assignment

Sequences were successfully obtained for all samples through DNA metabarcoding, followed by quality control filtering based on specific criteria from Trimmomatic v.0.36 [42]. This involved filtering out bases with a quality value below 20 at the end of the reads, using a 10 bp window to assess average quality, and truncating the back-end bases if the average quality within the window was below 20, while discarding reads below 100 bp. Paired sequences were then merged into a single sequence using FLASH (version 1.2.7) based on their overlap relation. Usearch software [43] (version 10, http://drive5.com/uparse/, accessed on 9 October 2023) was then employed to compare the sequences with reference sequences from the GOLD database (https://gold.jgi.doe.gov/, accessed on 9 October 2023), additional reference sequences, and de novo sequences to detect and remove chimeras. Additionally, primer removal was conducted using Cutadapt (v4.0, https://cutadapt.readthedocs.io/, accessed on 16 October 2023), followed by the analysis of high-quality sequences through molecular operational taxonomic unit (MOTU) clustering with a sequence similarity threshold of ≥99% using Usearch software. The comparison, classification, and annotation of MOTU representative sequences were performed against the MitoFish database (http://mitofish.aori.u-tokyo.ac.jp, accessed on 27 October 2023) and the NCBI database (http://www.ncbi.nlm.nih.gov/, accessed on 27 October 2023) using the Blastn tool (version 2.15.0)and the uclust algorithm [44]. MOTUs were further screened for fish species comparisons based on criteria of ≥97% similarity, e-value ≤ 10^–5^, and coverage ≥ 0.9, while non-fish sequences were excluded. Moreover, to assess the presence or absence of fish species in the basin, a historical list of fish species from the mainstem of the Jialing River was compiled using traditional survey methods such as drift gillnets, cast nets, ground cages, and purchase of fishermen’s captures. This historical list was then compared with the fish species identified using the eDNA method to exclude species not native to the basin [36,45,46,47] (Supplementary Table S1).

### 2.5. Species Composition and Diversity Analysis

To assess sequencing depth, dilution curves were generated using the Biozeron cloud platform (http://www.cloud.biomicroclass.com/CloudPlatform, accessed on 20 November 2023) [48]. To ensure uniformity among samples for inter-sample comparisons, reads from each sample were randomly selected using QIIME v.1.9.0, and the data from all eDNA samples were standardized based on the minimum number of samples sequenced across all samples [49]. The read ratio for each species in each sample remained consistent after standardization. The mean of three replicates was calculated for further analysis. All samples were categorized using two grouping methods based on geographical location—reservoir areas and the upper, middle, and lower reaches—for subsequent analysis. Species composition was analyzed using the results from the MOTU cluster analysis. A stacked histogram depicting the relative sequence abundance at the genus taxonomic level was created to visualize the fish species composition in each section of the river. Additionally, the ecological characteristics of the diverse fish species monitored were tallied (Supplementary Table S2), with stacked bar charts based on four ecotypes: flow rate, habitat layer, diet, and spawning type.

Furthermore, analyses of α-diversity and β-diversity were performed based on these findings. Alpha diversity, commonly measured as species richness, pertains to the variety of species within a confined area, reflecting the outcomes within the sample. Three indices were selected for evaluation: the Shannon index [50], the Simpson index [51], and the Pielou index [52]. The alpha diversity index was determined using the Biozeron cloud platform. Using appropriate tests (e.g., Kolmogorov–Smirnov test, Levene’s test) in SPSS, one-way analysis of variance (ANOVA) was then employed for data meeting these assumptions. For data violating these assumptions, the Kruskal–Wallis test was employed to evaluate differences in alpha diversity between samples. Beta diversity, which captures variations in species characteristics across different locations, was calculated to compare results between samples. The Jaccard distance and the Bray–Curtis matrix were employed for principal coordinate analysis (PCoA) based on the sequence numbers of fish species in each river section to assess the similarity in fish species composition among different river sections. The variation among groups was analyzed through permutational multivariate analysis of dispersion (PERMDISP) and permutation multivariate analysis of variance (PERMANOVA). For statistical analyses and visual representations, the vegan package and ggplot2 package in R software (version 4.0.3) were used (https://www.r-project.org/, accessed on 3 January 2024). The McNaughton index was used to identify the dominant species [53]. The flowing-water fish and small-bodied fish among the dominant species (*Y*_i_ > 0.02) in each river section were counted, and their proportions were calculated based on species numbers. The specific formulas used for calculating these indices are provided below:

Shannon index: *H*’ = ∑ *P*_i_log*P*i, *P*_i_ = n_i_/*N*

Simpson index: *D* = 1 − ∑ n_i_(n_i_ − 1)/*N*(*N* − 1)

Pielou index: *J* = *H*/*H*_max_

McNaughton index: *Y*_i_ = n_i_/*N* × f_i_

In the formulas, “*N*” represents the total number of detected fish sequences, and “n_i_” represents the number of sequences for the i-th fish species. “*H*” is the Shannon index. “*H*_max_” denotes the maximum Shannon index achievable under equal species richness (i.e., all species in the community have the same richness). *Y*_i_ represents the dominance index of the i-th fish species. f_i_ is the frequency of occurrence of the i-th species.

## 3. Results

### 3.1. Sequence Information and Annotation Results

Universal primers were successfully used in PCR amplification from 60 samples. The sequencing data have been deposited in the NCBI Sequence Read Archive (SRA) database under accession numbers SRR28122624-SRR28122683. After filtering low-quality sequences, removing chimeras, and eliminating non-fish sequences, 15,599,223 valid sequences were obtained (the sequence count for each sample before and after filtration is shown in Supplementary Table S3), resulting in 170 MOTUs through clustering. Following manual screening, 99 MOTUs were identified and annotated to 99 fish species, representing 74 genera, 7 orders, and 20 families (*Acipenser* species were not identified at the species level) (Supplementary Table S4). Dilution curves constructed based on the quantity of sequencing data showed all curves reaching a plateau, confirming adequate sequencing depth for all samples (Supplementary Figure S1).

### 3.2. Diversity in Fish Composition

**Figure 2:**
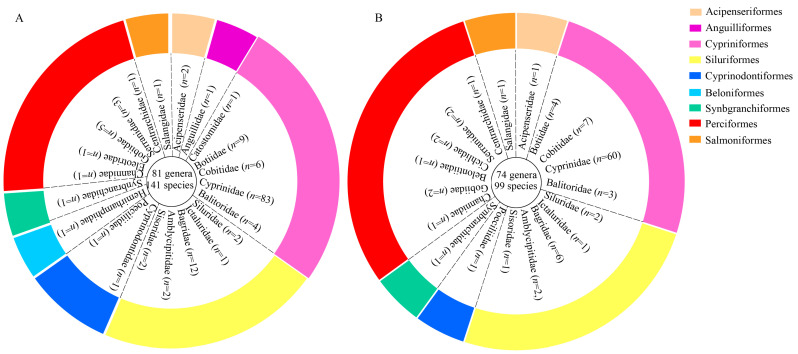
(**A**) Proportion of species at the order level in historical fish and (**B**) eDNA monitoring results (the center of the circle represents the genera and species detected by the method. The “*n*” represents the number of species within that family).

Over the past two decades (2003–2023), a total of 141 fish species have been documented in the mainstem basin of the Jialing River spanning 81 genera across 9 orders and 22 families (Supplementary Table S1). Among these, 9 nationally protected fish species, 32 endemic species from the upper reaches of the Yangtze River, and 11 alien species have been identified. Notably, the families Cyprinidae, Bagridae, and Botiidae exhibit the highest proportions of species numbers, representing 58.87%, 8.51%, and 6.38% of the total species count, respectively. Other families are represented by fewer species (Figure 2A).

**Figure 3:**
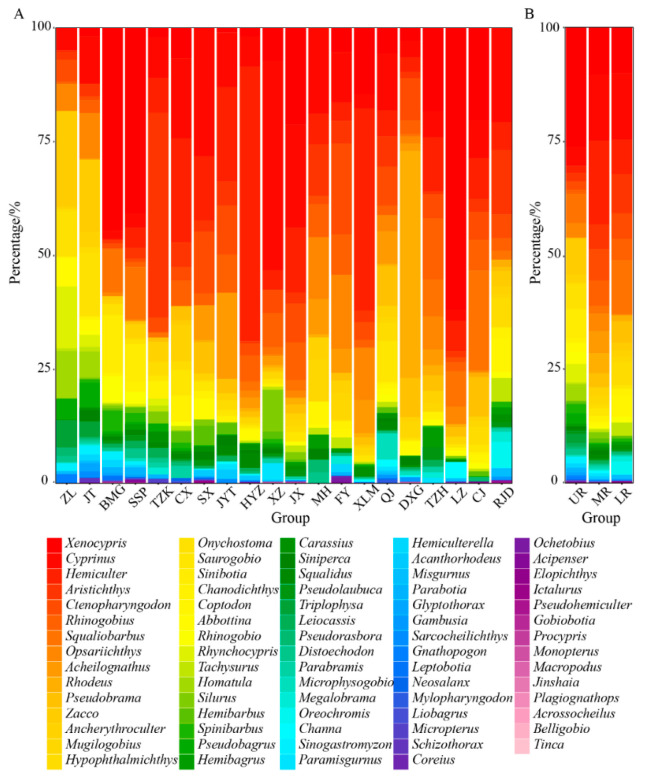
Stacked histogram based on sequence abundance at the genus level: (**A**) reservoir areas (The abbreviation’s meaning refers to the figure caption of Figure 1); (**B**) UR: the upper reaches of the Jialing River; MR: the middle reaches of the Jialing River; LR: the lower reaches of the Jialing River. Different colors represent various fish genera.

Through eDNA analysis, a total of 99 fish species were detected (with *Acipenser* not identified at the species level). Among these, 5 nationally protected species, 18 endemic fish species from the upper reaches of the Yangtze River, and 11 alien fish species were identified (Supplementary Table S4). Cyprinidae displayed the highest species count with 60 species (60.61%), followed by Cobitidae with 7 species (7.07%) and Bagridae with 6 species (6.06%) (Figure 2B). Notably, genera such as *Xenocypris*, *Cyprinus*, and *Hemiculter* exhibited extremely high relative sequence abundance, whereas species of *Jinshaia*, *Acrossocheilus*, and *Macropodus* accounted for a relatively low proportion of sequence abundance (Figure 3).

Of the 99 species detected by eDNA, 88 species were native fishes, comprising 88.89%, while 11 species were alien fishes, accounting for 11.11% (Supplementary Table S4). Among these, all species were previously recorded in historical data except for three: *Oreochromis niloticus*, *Coptodon zillii*, and *Pseudohemiculter dispar*. Notably, *C. zillii* has emerged as a dominant species in the RJD and LR regions, while the relative sequence abundance of *O. niloticus* remains relatively high in the RJD.

### 3.3. Diversity of Fish Communities

#### 3.3.1. Ecotype Composition

**Figure 4:**
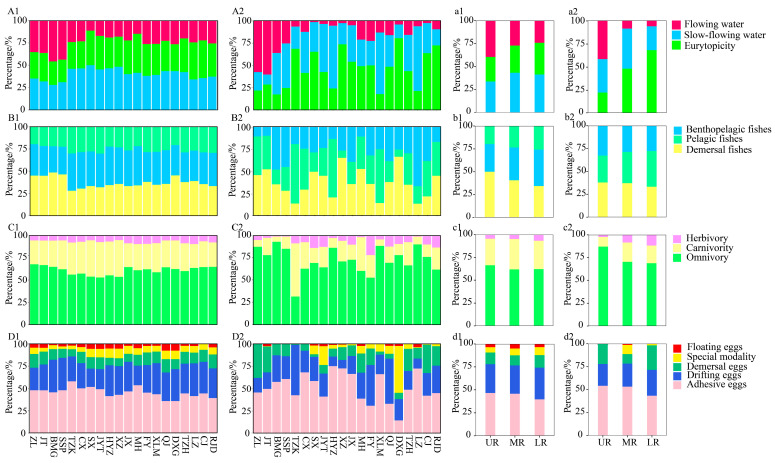
The proportion of each ecotype based on species numbers (**A1**,**a1**,**B1**,**b1**,**C1**,**c1**,**D1**,**d1**) and the proportion of each ecotype based on fish sequence numbers (**A2**,**a2**,**B2**,**b2**,**C2**,**c2**,**D2**,**d2**) (**A**,**a**: flow rate; **B**,**b**: habitat layer; **C**,**c**: diet; **D**,**d**: spawning type).

In terms of species presence, the fish ecotypes in the river sections, UR, MR, and LR, all of which were dominated by slow-flowing water, demersal fish, omnivores, and fish spawning adhesive eggs, exhibited similar compositions (Figure 4). However, the UR section (including the ZL, JT, BMG, SSP reservoirs) exhibited a higher diversity of fish favoring flowing water habitats. Based on relative sequence abundance, the composition of fish ecotypes showed minimal variation across reservoir areas, UR, MR, and LR. Overall, there was a higher proportion of eurytopic species, demersal fish, omnivores, and adhesive-egg spawners (Figure 4). In terms of flow rate preferences, fish favoring flowing water had a lower relative sequence abundance compared to eurytopic fish. Demersal fish showed the highest relative sequence abundance in the habitat layer, while benthopelagic fish displayed the lowest. Regarding spawning type, fish that spawn floating eggs had the lowest relative sequence abundance, comprising only 1.04%, whereas adhesive-egg spawning species accounted for the highest proportion at 52.87%. Overall, there was no significant difference (*p* > 0.05) in the composition of fish ecotypes in each reservoir area, UR, MR, and LR.

#### 3.3.2. Dominant Species

Across all groups, less than 50% of fish species in most river sections, except for the ZL and BMG reservoirs, and the UR segment, preferred flowing water habitats (Supplementary Table S5). Among the species with dominant sequence, small-bodied fish such as *Hemiculter leucisculus*, *H. tchangi*, *Rhinogobius cliffordpopei*, *Mugilogobius myxodermus*, and *Abbottina rivularis* were discovered (fish with an adult body length of <20 cm were defined as small-bodied). In most river segments, the proportion of small-bodied fish was ≥50%, except for the SSP, TZK, CX, RJD, and LR segments (Supplementary Table S5).

#### 3.3.3. Analysis of Alpha and Beta Diversity in Each River Section

**Figure 5:**
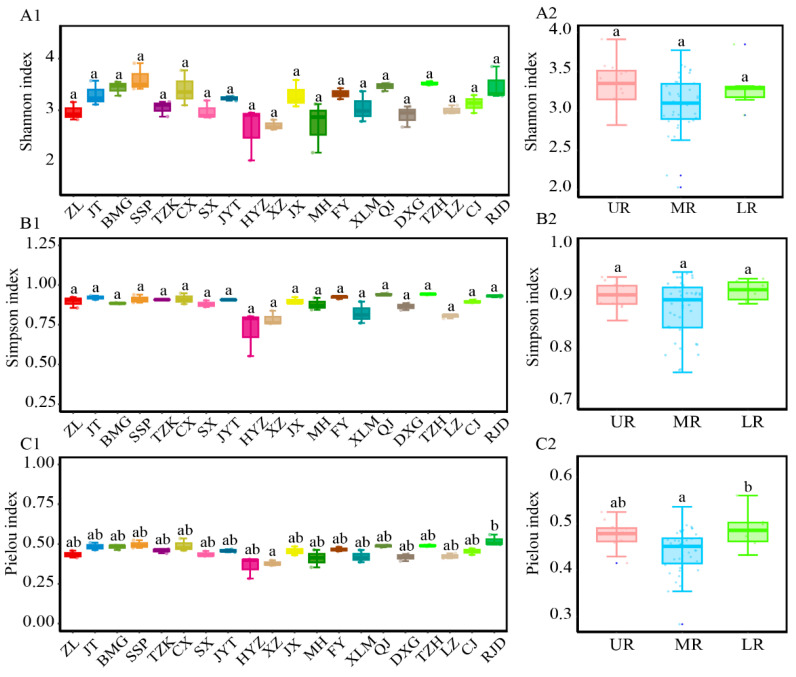
Box plots of alpha diversity by reservoir grouping (**A1**–**C1**) and by grouping in upper, middle, and lower reaches (**A2**–**C2**). The absence of the same letter (a, ab, b) indicates a significant difference.

Among all river sections, the HYZ reservoir displayed the lowest Shannon, Simpson, and Pielou indices, while the SSP reservoir had the highest Shannon index; the TZH reservoir had the highest Simpson index, and the RJD showed the highest Pielou index (Figure 5A1–C1). The Pielou index in the RJD was significantly higher than that in the XZ reservoir (df = 19, *p* = 0.046). However, no significant differences (*p* > 0.05) were observed in the Shannon or Simpson indices across all river sections. For the upper, middle, and lower reaches (Figure 5A2–C2), the highest Shannon and Simpson indices were observed in the UR, whereas all three alpha diversity indices were the lowest in the MR. The highest Pielou index was observed in the LR. The Pielou index in the LR showed a significant increase (df = 2, *p* = 0.041) compared to that in the MR, while the Shannon and Simpson indices did not show significant differences (*p* > 0.05).

**Figure 6:**
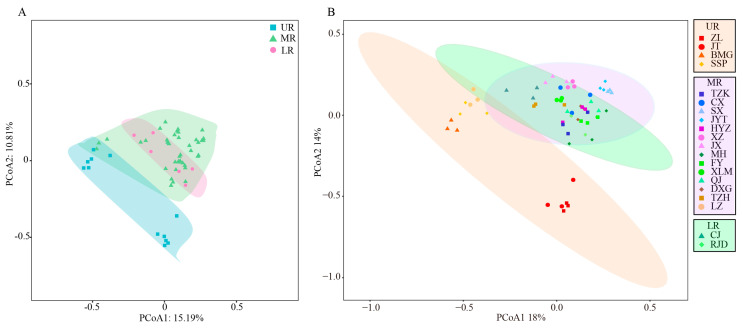
The Jaccard distance (**A**) and the Bray–Curtis matrix (**B**) were employed for PCoA based on the sequence numbers of fish species in each river section.

The PERMDISP analysis indicated no significant differences between groups (F = 2.307, *p* = 0.19). However, the PERMANOVA analysis revealed significant differences among the three river sections (R^2^ = 0.2704, *p* = 0.001). The PCoA based on Jaccard distance explained 15.19% of the total variation along the first axis and 10.81% along the second axis (Figure 6A). Similarly, the PCoA utilizing the Bray–Curtis distance accounted for 18% and 14% of the variation along the two axes, respectively (Figure 6B). In both PCoAs, the confidence intervals for the UR showed minimal overlap with those of the MR and LR, indicating distinct differences. In contrast, there was considerable overlap in the confidence intervals between the MR and LR, suggesting higher similarity between these two sections (Figure 6).

## 4. Discussion

### 4.1. Diversity of Fish Composition

Significantly fewer fish species were detected in the mainstem of the Jialing River compared to in historical records. This study identified only 99 fish species, whereas 141 fish species have been recorded in the basin over the past two decades. The absence of species such as *Micropercops swinhonis*, *Myxocyprinus asiaticus*, *Anguilla japonica*, and *Oryzias latipes*—previously recorded in historical surveys—may be attributed to their decline or disappearance due to increased anthropogenic activities. Some fish species detected in this study, although not caught in the past 20 years, were previously recorded in the basin. This might be due to traditional fishing nets failing to capture these fishes or their absence from the specific fishing locations surveyed. Moreover, the detection of three fish species exclusively through eDNA analysis, which were not recorded in historical data, indicates that they are alien species. This suggests that these alien fishes could have spread to the basin in recent years, potentially escaping from nearby aquaculture farms into the river. These findings highlight the feasibility and effectiveness of the eDNA method for monitoring fish diversity in the Jialing River. It serves as a supplementary tool to traditional survey methods, enhancing the ability to detect both native and alien fish species [40].

The modification of habitat hydrological conditions due to anthropogenic activities, especially dam construction, may create a more favorable environment for alien fish while posing significant challenges to the adaptation and survival of native fish [54,55]. This disparity in habitat suitability can lead to a competitive disadvantage for native fish in accessing spawning grounds, food resources, and other essential elements for their survival [56]. Studies have demonstrated that alien fishes often possess strong survival and reproductive capabilities, which can disrupt the habitat and distribution of native fish species, ultimately resulting in a decline or even extinction of native fish populations [36]. Additionally, alien fishes such as *M. salmoides* and *Ietalurus punetaus* prey on smaller native fish, as well as their fry and eggs, further altering the species composition of native fish community [36,57]. The presence of alien fishes is largely driven by anthropogenic activities, including escape from aquaculture farms and indiscriminate release [36,58]. Consequently, the introduction of alien fishes into the Jialing River basin is likely attributed to these anthropogenic activities, posing an ongoing threat to native fish populations [58].

### 4.2. Diversity of Fish Communities

The abundant fish resources of the Jialing River are intricately linked to the distinctive water environment of the Jialing River Basin, where fast-flowing shoals serve as principal habitats for flowing water fish [45]. Nevertheless, anthropogenic activities, particularly dam construction, have disturbed these habitats and altered the river’s ecosystem, including flow, depth, and velocity, thus transforming it from a flowing water environment to a semi-lentic water environment [59]. Our data indicate that these changes have led to a significantly lower abundance of fish preferring flowing water in the MR and LR compared to the UR. This disparity may be associated with the longer length and greater elevation drop in the UR reservoirs, which offer more suitable habitats for flowing-water fish, resulting in a higher proportion of sequence numbers for these species. Certain genera, such as *Cobitis* and *Parabotia*, which are adapted to flowing water habitats, have experienced declines in the mainstem of the Jialing River. Conversely, genera like *Acheilognathus* and *Xenocypris*, which thrive in semi-lentic or eurytopic environments, have been able to persist. Dams can affect the genetic diversity of some fish populations. For example, reductions in habitats may result in some fish species that prefer flowing water and spawn drifting eggs being unable to adapt to the changed environment, and populations of these species may continue to decline which may result in the loss of some genotypes and a decrease in genetic diversity [60,61]. Additionally, dam construction has deepened the rivers, increased sediment deposition, and enhanced the availability of demersal bait organisms, creating more favorable conditions for demersal fish [62].

Analysis of the eDNA monitoring data indicated that eurytopicity, demersal fish, and spawning adhesive eggs dominated the ecotype composition across different river sections (UR, MR, and LR), as determined by relative sequence abundance of the fish. Eurytopic fish, known for their adaptability and resilience to environmental changes, were prevalent throughout all river segments [63]. The main channel of the Jialing River, characterized by abundant hydrophytes and a substrate predominantly composed of gravel, sand, and mud, provides a favorable environment for the reproduction of fish that spawn adhesive eggs. The majority of Cyprinidae exhibit adhesive-egg spawning behavior, rendering them more resilient to environmental changes associated with anthropogenic activities. In conclusion, the ecological changes resulting from dam construction align with the predominant ecological traits of the fish species detected, highlighting the adaptability of certain species to altered habitats.

The eDNA monitoring results for dominant fish species indicated that the Jialing River mainstem basin has been predominantly inhabited by small-bodied fish, with a relatively low proportion of sequence-dominant fish species preferring flowing water habitats. Overfishing in the Yangtze River Basin prior to the fishing ban could have contributed to this phenomenon. Additionally, the modification of fish habitats due to anthropogenic activities, such as river channel hardening, sand mining, and dam construction, might also play a role in the lack of suitable conditions for the unrestricted movement, reproduction, and feeding of medium and large fish, thereby hindering the completion of a full life cycle for juvenile fish [64,65] and reducing the availability of flowing water habitats preferred by certain fish species. These findings align with research conducted in various regions of the Yangtze River basin, including the middle reaches of the Jialing River, the mainstem of the Wujiang River, and the Three Gorges Reservoir [34,58,66].

Studies have demonstrated that anthropogenic activities can reduce fish population sizes and alter fish species composition [67]. Across all river sections, the highest Shannon and Simpson index values were observed in the UR (particularly the SSP reservoir area in the UR, which had the highest Shannon index among all reservoirs), indicating greater community diversity in this region. This could be attributed to the longer length of the reservoirs in the UR and the lesser impact of terraced hydropower developments on the original river habitat compared to other reaches [20]. In contrast, the lowest Shannon, Simpson, and Pielou indices were recorded in the MR, specifically in the HYZ reservoir. The reduced community diversity in this section may be due to the disproportionately high relative sequence abundance of *H. leucisculus*, *R. cliffordpopei*, *Squalidus argentatus*, *Cyprinus carpio*, and other fishes [50]. This uneven species distribution likely contributed to a decline in α-diversity. Additionally, most reservoirs in the MR are shorter than 40 km in length, intensifying the influence caused by terrace development. Significant differences in Pielou indices between the MR and LR (as well as notable differences in Pielou indices between the XZ reservoir in the MR and the RJD reservoir in the LR) were likely due to the exceptionally high relative sequence abundance of individual species, resulting in significant differences in evenness. However, overall, there were no significant differences (*p* < 0.05) in alpha indices among the terraced reservoirs or between the UR, MR, and LR.

The fish community composition in the MR and LR of the Jialing River showed similarities, as indicated by the PCoA. Contrarily, the UR displayed distinct differences from the MR and LR. Studies have shown that changes in the hydrological regime of rivers can affect fish population sizes and genetic diversity [68]. The UR is characterized by alpine environments with narrow valleys, fast-flowing water, significant altitude variations, and limited human interference. The MR and UR sections feature broad, semi-lentic environments with minimal altitude changes and high levels of urbanization. These river sections in the MR and LR are located near parks, marinas, and dredging and quarrying sites and experience heavy shipping activity downstream of Guangyuan, making them more susceptible to human impacts [36]. Consequently, noticeable disparities in fish community composition were observed between the UR and MR, LR. The similarity in fish composition between the MR and LR can be attributed to their comparable habitat conditions and similar tributary environments [69,70]. These findings imply that fish community composition in the Jialing River is shaped by a combination of natural factors, such as altitude and flow dynamics, and anthropogenic factors, including urbanization and industrial activities.

Anthropogenic activities, especially dam construction, have been proposed as significant contributors to the homogenization of freshwater ecosystems, leading to habitat uniformity [71,72,73]. This homogenization may stem from the fragmentation or destruction of natural river features such as rapids and shallows caused by dams, leading to reduced habitat heterogeneity and a corresponding decline in the diversity of fish species adapted to various habitats. Additionally, the introduction and spread of alien fish species can further intensify the decline or extinction of native fish populations through predation and competition, ultimately decreasing beta diversity and accelerating biological homogenization [74,75]. As such, the observed similarity in fish composition between the MR and LR sections of the Jialing River indicated an ongoing process of ecological homogenization.

### 4.3. Limitations of eDNA Technology

Although eDNA technology is a promising tool widely embraced by researchers, certain uncertainties persist in the research process [76,77], such as the susceptibility of eDNA to degradation. Temperature, a significant abiotic factor, affects the concentration of eDNA, with higher temperatures leading to increased shedding and decay rates [78,79]. To mitigate these effects, water samples were promptly stored in a cold environment using foam boxes and ice packs for cryopreservation, and filtration was conducted on the same day. Additionally, the possibility of false-positive and false-negative results must be acknowledged. While certain fish species may be detected, there is no guarantee that they actually exist at the sampling site, as their eDNA could originate from sources such as domestic water or animal feces. However, the concentration of eDNA was minimal and had little impact on our results [80]. Moreover, we mitigated the limitations of eDNA technology by enhancing the quality of annotation and compiling a comprehensive list of historically documented fish species in the basin to assess their presence or absence. The absence of detected fish could be attributed to the spatiotemporal variations in fish populations. For future research, a more comprehensive approach involving a broader sampling scope, a higher sampling frequency, and a greater number of replicates would be essential to improve accuracy and reliability [81].

### 4.4. Measures to Conserve Fish Diversity

Anthropogenic activities can have both beneficial and adverse impacts on river ecosystems. Among these actions, terraced development offers various benefits, such as flood control, agricultural irrigation, and energy supply, yielding substantial economic advantages. However, it can also result in hydrological changes, biodiversity loss, and negative impacts on freshwater ecosystems [82,83]. This study revealed a convergence in fish diversity across the 20 river sections, characterized by a predominance of small-bodied fish, and a reduced presence of fish preferring flowing water. In light of these findings and the observed spatial state of fish diversity in the Jialing River mainstem in the context of anthropogenic activities, measures such as enhancing habitat protection, establishing local nature reserves, implementing artificial breeding and hatchery release programs for endangered species, and regulating angling activities can be employed to protect fish populations. Previous studies have shown the effectiveness of these conservation efforts in facilitating the gradual recovery of fishery resources [84,85,86].

## 5. Conclusions

eDNA technology, as demonstrated in this study, proves to be a valuable tool for assessing biodiversity and understanding the effects of anthropogenic activities on fish species composition and diversity in the Jialing River mainstem. This study found that Cyprinidae fish dominate in the highest proportion. The ecotypes of the fish community are predominantly characterized by species that prefer slow-flowing water, demersal fish, omnivores, and fish spawning adhesive eggs. Furthermore, small-bodied fish dominate the fish sequences in the Jialing River mainstem, with a relatively low proportion of fish preferring flowing habitats. Geographic zoning analysis revealed minimal disparities in fish species composition and species diversity among the terraced reservoirs, as well as between the upper, middle, and lower reaches of the river. Notably, the fish compositions in the middle and lower reaches were highly similar, suggesting a trend of homogenization in these areas. These findings underscore the potential of the eDNA method as a valuable supplementary tool for monitoring fishery resources in the Jialing River Basin.

## Data Availability

The reference sequences are available in the NCBI (https://dataview.ncbi.nlm.nih.gov) Sequence Read Archive (SRA) database under the following accession numbers: SRR28122624-SRR28122683.

## Supplementary Materials

The supplementary materials can be downloaded at: https://www.mdpi.com/article/10.3390/ani15010105/s1, Figure S1: Dilution curves. The Shannon index of each environmental DNA’s PCR products increases with the sequence reads; Table S1: List of historical fish species in the main stream of the Jialing River by traditional methods; Table S2: Ecotypes of fish monitored by eDNA technology; Table S3: The sequence count per sample before and after filtration; Table S4: The eDNA technology detected fish species and their taxonomic status, as well as the sequence numbers of these fish detected in each sampling area; Table S5 The dominant sequence species in each river section. Additionally, count the total number of dominant fish species in each river sections and calculate the proportion of fish preferring flowing water and small-bodied fish among them.
