# Supplementary material for: Environmental DNA Insights into the Spatial Status of Fish Diversity in the Mainstem of the Jialing River
Source: Animals (Basel). 2025 Jan 5;15(1):105. doi: 10.3390/ani15010105 (PMC11718828; doi:10.3390/ani15010105)
Supplement: Supplementary file 1 [file animals-15-00105-s001.zip › animals-3351292-supplementary.pdf]

Supplementary Figure S1 Dilution curves. The Shannon index of each environmental DNA's PCR products increases with the sequence reads

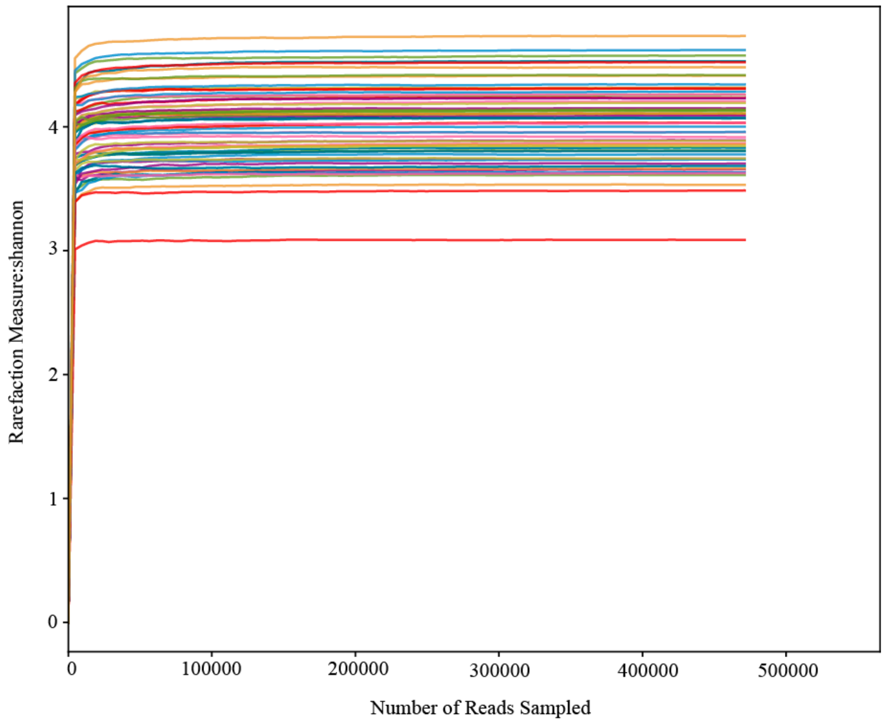

Supplementary Table S1 List of historical fish species in the main stream of the Jialing River by traditional methods

Serial number	Order	Family	Genus	Specie	Fish collected in the last 20 years	The endemic fish of the upper Yangtze River	Nationally protected fish
1	Acipenseriformes	Acipenseridae	*Acipenser*	*A. dabryanus*	+	+	+
2				*A. sinensis*	+		+
3		Polyodontidae	*Psephurus*	*P. gladius*			+
4	Anguilliformes	Anguillidae	*Anguilla*	*A. japonica*	+		
5	Cypriniformes	Catostomidae	*Myxocyprinus*	*M. asiaticus*	+		+
6		Botiidae	*Parabotia*	*P. fasciatus*	+		
7				*P. bimaculata*	+	+	
8			*Sinibotia*	*S. superciliaris*	+		
9				*S. reevesae*	+	+	
10			*Leptobotia*	*L. elongata*	+	+	+
11				*L. taeniaps*	+		
12				*L. pellegrini*	+		
13				*L. microphthalrna*	+	+	

14				*L. rubrilabris*	+	+	+
15		Cobitidae	*Triplophysa*	*T. robusta*			
16				*T. orientalis*			
17				*T. brevviuda*			
18				*T. bleekeri*	+	+	
19				*T. stoliczkae*			
20				*T. stenura*			
21				*T. obscura*			
22				*T. pappenheimi*			
23			*Homatula*	*H. variegata*	+		
24				*H. potanini*	+	+	
25			*Cobitis*	*C. sinensis*	+		
26			*Misgurnus*	*M. anguillicaudatus*	+		
27			*Paramisgurnus*	*P. dabryanus*	+		
28			*Claea*	*C. dabryi*		+	
29		Cyprinidae	*Zacco*	*Z. platypus*	+		
30			*Opsariichthys*	*O. bidens*	+		
31				*O. macrolepis*			
32			*Aphyocypris*	*A. chinensis*			
33			*Ctenopharyngodon*	*C. idella*	+		
34			*Luciobrama*	*L. macrocephalus*	+		
35			*Mylopharyngodon*	*M. piceus*	+		
36			*Squaliobarbus*	*S. curriculus*	+		
37			*Rhynchocypris*	*R. lagowskii*			
38			*Tinca*	*T. tinca**	+		
39			*Elopichthys*	*E. bambusa*	+		
40			*Ochetobius*	*O. elongatus*	+		
41			*Hemiculterella*	*H. sauvagei*	+	+	
42			*Hemiculter*	*H. leucisculus*	+		
43				*H. tchangi*	+	+	
44				*H. bleekeri*	+		
45				*H. nigromarginis*	+		
46			*Ancherythroculter*	*A. kurematsui*	+	+	
47				*A. wangi*		+	
48				*A. nigrocauda*	+	+	
49			*Pseudolaubuca*	*P. sinensis*	+		
50				*P. engraulis*	+		
51			*Chanodichthys*	*C. erythropterus*	+		
52				*C. alburnus*	+		
53				*C. mongolicus*	+		
54				*C. oxycephalus*	+		

55		*C. oxycephaloides*	+		
56		*C. dabryi*	+		
57	*Megalobrama*	*M. amblycephala**	+		
58		*M. terminalis**	+		
59		*M. pellegrini*	+	+	
60	*Xenocypris*	*X. davidi*	+		
61		*X. argentea*	+		
62		*X. fangi*	+	+	
63		*X. yunnanensis*	+		
64		*X. sechuanensis*		+	
65		*X. macrolepis*	+		
66	*Plagiognathops*	*P. microlepis*	+		
67	*Distoechodon*	*D. tumirostris*	+		
68	*Parabramis*	*P. pekinensis*	+		
69	*Pseudobrama*	*P. simoni*	+		
70	*Pseudogobio*	*P. vaillanti*	+		
71	*Sinibrama*	*S. wui*			
72		*S. taeniatus*	+	+	
73	*Acheilognathus*	*A. macropterus*	+		
74		*A. omeiensis*	+	+	
75		*A. gracilis*	+		
76		*A. barbatulus*	+		
77	*Acanthorhodeus*	*A. chankaensis*			
78	*Paracheilognathus*	*P. imberbis*	+		
79	*Rhodeus*	*R. sinensis*	+		
80		*R. lighti*	+		
81		*R. ocellatus*	+		
82	*Luciobarbus*	*L. capito**	+		
83	*Spinibarbus*	*S. hollandi*			
84		*S. caldwelli*			
85		*S. sinensis*	+	+	
86	*Acrossocheilus*	*A. yunnanensis*	+		
87		*A. monticola*	+	+	
88	*Onychostoma*	*O. macrolepis*	+		+
89		*O. simum*	+		
90		*O. angustistomata*		+	+
91	*Hemibarbus*	*H. maculatus*	+		
92		*H. labeo*	+		
93	*Belligobio*	*B. nummifer*			
94	*Folifer*	*F. brevifilis*	+		
95	*Bangana*	*B. rendahli*	+	+	

96		*Pseudorasbora*	*P. parva**	+		
97		*Sarcocheilichthys*	*S. sinensis*	+		
98			*S. nigripinnis*	+		
99		*Gnathopogon*	*G. herzensteini*	+	+	
100			*G. imberbis*	+		
101		*Coreius*	*C. heterodon*	+		
102			*C. guichenoti*	+	+	+
103		*Squalidus*	*S. argentatus*	+		
104			*S. wolterstorffi*	+		
105		*Rhinogobio*	*R. typus*	+		
106			*R. cylindricus*	+	+	
107			*R. ventralis*	+	+	+
108		*Platysmacheilus*	*P. nudiventris*		+	
109		*Abbottina*	*A. rivularis*	+		
110			*A. obtusirostris*	+	+	
111		*Microphysogobio*	*M. kiatingensis*			
112		*Saurogobio*	*S. dabryi*	+		
113			*S. dumerili*			
114			*S. gymnocheilus*	+		
115			*S. punctatus*	+		
116		*Gobiobotia*	*G. filifer*	+		
117		*Xenophysogobio*	*X. boulengeri*	+	+	
118		*Schizothorax*	*S. sinensis*	+	+	
119			*S. prenanti*	+	+	
120			*S. chongi*		+	+
121			*S. davidi*		+	+
122		*Gymnodiptychus*	*G. pachycheilus*			+
123		*Schizopygopsis*	*S. kialingensis*		+	
124		*Carassius*	*C. auratus*	+		
125		*Cyprinus*	*C. carpio*	+		
126			*C. carpio L. mirror**	+		
127		*Procypris*	*P. rabaudi*	+	+	+
128		*Hypophthalmichthys*	*H. molitrix*	+		
129		*Aristichthys*	*A. nobilis*	+		
130	Balitoridae	*Lepturichthys*	*L. fimbriata*	+		
131		*Jinshaia*	*J. sinensis*	+	+	
132			*J. abbreviata*		+	
133		*Hemimzon*	*H. yaotanensis*		+	
134		*Sinogastromyzon*	*S. szechuanensis*	+	+	
135			*S. sichangensis*		+	
136		*Metahomaloptera*	*M. omeiensis*	+	+	

137	Siluriformes	Siluridae	*Silurus*	*S. asotus*	+		
138				*S. meridionalis*	+		
139		Ictaluridae	*Ictalurus*	*I. punctatus**	+		
140		Bagridae	*Pseudobagrus*	*P. pratti*	+		
141				*P. brevicaudatus*	+		
142				*P. truncatus*	+		
143				*P. emarginatus*	+		
144				*P. ussuriensis*	+		
145			*Tachysurus*	*T. fulvidraco*	+		
146				*T. eupogon*	+		
147				*T. vachelli*	+		
148				*T. nitidus*	+		
149			*Leiocassis*	*L. dumerili*	+		
150				*L. crassilabris*	+		
151			*Hemibagrus*	*H. macropterus*	+		
152		Amblycipitidae	*Liobagrus*	*L. marginatus*	+		
153				*L. nigricauda*	+		
154		Sisoridae	*Pareuchiloglanis*	*P. anteanalis*		+	
155			*Glyptothorax*	*G. sinensis*	+		
156				*G. fokiensis*	+		
157			*Euchiloglanis*	*E. davidi*		+	+
158				*E. kishinouyei*		+	
159	Cyprinodontiformes	Cyprinodontidae	*Oryzias*	*O. latipes*	+		
160		Poeciliidae	*Gambusia*	*G. affinis**	+		
161	Beloniformes	Hemirhamphidae	*Hyporhamphus*	*H. intermedius**	+		
162	Synbgranchiformes	Synbranchidae	*Monopterus*	*M. albus*	+		
163	Perciformes	Channidae	*Channa*	*C. argus*	+		
164		Eleotridae	*Micropercops*	*M. swinhonis*	+		
165		Gobiidae	*Rhinogobius*	*R. giurinus*	+		
166				*R. brunneus*			
167				*R. szechuanensis*	+	+	
168				*R. cliffordpopei*	+		
169				*R. leavelli*	+		
170			*Mugilogobius*	*M. myxodermus*	+		
171		Belontiidae	*Macropodus*	*M. chinensis*			
172				*M. opercularis*			
173		Serranidae	*Siniperca*	*S. chuatsi*	+		
174				*S. scherzeri*	+		
175				*S. knerii*	+		

176		Centrarchidae	*Micropterus*	*M. salmoides**	+		
177	Salmoniformes	Salangidae	*Neosalanx*	*N. taihuensis**	+		
Total	9	25	96	177	141	46	16

Note: +: meet the criterion, *: alien fish, ●: The endemic fish of the upper Yangtze River, ★: Nationally protected fish

Supplementary Table S2 Ecotypes of fish monitored by eDNA technology

Serial number	Order	Family	Genus	Species	Ecotype				UR	MR	LR
1	Acipenseriformes	Acipenseridae	*Acipenser*	*A.* sp.	R	B	O	A	+	+	
2	Cypriniformes	Botiidae	*Parabotia*	*P. fasciatus*	R	B	O	F	+	+	
3			*Sinibotia*	*S. superciliaris*	R	B	O	F	+	+	
4				*S. reevesae*●	R	B	O	F	+		
5			*Leptobotia*	*L. elongata*★●	R	B	O	A	+	+	
6		Cobitidae	*Triplophysa*	*T. orientalis*	R	B	O	D	+		
7				*T. bleekeri*●	R	B	O	D	+		
8				*T. stenura*	R	B	O	D	+		
9			*Homatula*	*H. variegata*	R	B	O	D	+		
10				*H. potanini*●	R	B	O	D	+		
11			*Misgurnus*	*M. anguillicaudatus*	G	B	O	A	+	+	+
12			*Paramisgurnus*	*P. dabryanus*	N	B	O	D	+	+	+
13		Cyprinidae	*Zacco*	*Z. platypus*	R	U	O	D	+	+	+
14			*Opsariichthys*	*O. bidens*	R	U	O	F	+	+	+
15			*Ctenopharyngodon*	*C. idella*	G	L	H	F	+	+	+
16			*Mylopharyngodon*	*M. piceus*	R	L	C	F	+	+	+
17			*Squaliobarbus*	*S. curriculus*	G	U	O	D	+	+	+
18			*Rhynchocypris*	*R. lagowskii*	N	U	O	A	+	+	
19			*Tinca*	*T. tinca**	N	B	O	A	+		
20			*Elopichthys*	*E. bambusa*	G	U	C	F		+	+
21			*Ochetobius*	*O. elongatus*	N	B	C	F	+	+	+
22			*Hemiculterella*	*H. sauvagei*●	R	U	O	D	+	+	+
23			*Hemiculter*	*H. leucisculus*	N	U	O	A	+	+	+
24				*H. tchangi*●	N	U	O	A	+	+	+
25			*Pseudohemiculter*	*P. dispar**	N	U	O	A	+	+	+
26			*Ancherythroculter*	*A. wangi*●	R	U	C	A	+	+	+
27			*Pseudolaubuca*	*P. sinensis*	G	U	O	F	+	+	+
28				*P. engraulis*	G	U	O	F	+	+	
29			*Chanodichthys*	*C. erythropterus*	N	U	C	A	+	+	
30				*C. alburnus*	N	U	C	A	+	+	+

31		*C. oxycephaloides*	N	U	C	F	+	+	+
32		*C. dabryi*	N	U	C	A	+	+	+
33	*Megalobrama*	*M. amblycephala**	N	L	H	A	+	+	+
34		*M. terminalis**	N	L	O	A	+	+	+
35	*Xenocypris*	*X. davidi*	G	L	O	A	+	+	+
36		*X. yunnanensis*	N	L	O	A	+	+	+
37	*Plagiognathops*	*P. microlepis*	N	L	O	A	+	+	+
38	*Distoechodon*	*D. tumirostris*	N	L	O	F	+	+	+
39	*Parabramis*	*P. pekinensis*	G	L	H	A	+	+	+
40	*Pseudobrama*	*P. simoni*	G	L	O	F	+	+	+
41	*Acheilognathus*	*A. macropterus*	N	B	O	S	+	+	+
42		*A. omeiensis*●	N	B	O	S		+	
43	*Acanthorhodeus*	*A. chankaensis*	N	L	O	S	+	+	+
44	*Rhodeus*	*R. sinensis*	G	L	O	S	+	+	+
45		*R. ocellatus*	G	B	O	S	+	+	+
46	*Spinibarbus*	*S. caldwelli*	R	L	O	F		+	
47		*S. sinensis*●	R	B	O	F	+	+	+
48	*Acrossocheilus*	*A. monticola*●	R	B	O	A	+		
49	*Onychostoma*	*O. macrolepis*★	R	B	O	A	+	+	
50	*Hemibarbus*	*H. maculatus*	N	L	C	A	+	+	+
51		*H. labeo*	N	B	C	A	+	+	+
52	*Belligobio*	*B. nummifer*	R	B	C	D		+	
53	*Pseudorasbora*	*P. parva**	G	L	O	A	+	+	+
54	*Sarcocheilichthys*	*S. sinensis*	N	L	O	A		+	
55		*S. nigripinnis*	G	L	O	F	+	+	
56	*Gnathopogon*	*G. herzensteini*●	R	L	O	F	+		+
57	*Coreius*	*C. heterodon*	R	B	O	F			+
58		*C. guichenoti*★●	R	B	O	F	+	+	
59	*Squalidus*	*S. argentatus*	R	L	O	F	+	+	+
60		*S. wolterstorffi*	R	L	O	F	+	+	+
61	*Rhinogobio*	*R. typus*	R	B	O	F	+	+	+
62		*R. cylindricus*●	R	B	O	F	+	+	+
63	*Abbottina*	*A. rivularis*	G	B	O	A	+	+	
64	*Microphysogobio*	*M. kiatingensis*	R	B	O	F	+	+	+
65	*Saurogobio*	*S. dabryi*	N	L	O	F	+	+	+
66	*Gobiobotia*	*G. filifer*	R	B	C	F	+		
67	*Schizothorax*	*S. davidi*★●	R	B	C	A	+		
68	*Carassius*	*C. auratus*	G	L	O	A	+	+	+
69	*Cyprinus*	*C. carpio*	G	B	O	A	+	+	+
70	*Procypris*	*P. rabaudi*★●	R	B	O	A		+	
71	*Hypophthalmichthys*	*H. molitrix*	G	U	H	F	+	+	+

72			*Aristichthys*	*A. nobilis*	G	U	H	F	+	+	+
73		Balitoridae	*Jinshaia*	*J. abbreviata*●	R	B	O	F	+		
74			*Sinogastromyzon*	*S. szechuanensis*●	R	B	O	A	+		
75				*S. sichangensis*●	R	B	O	F	+		
76	Siluriformes	Siluridae	*Silurus*	*S. asotus*	G	L	C	A	+	+	
77				*S. meridionalis*	G	L	C	A	+	+	+
78		Ictaluridae	*Ictalurus*	*I. punctatus**	G	B	O	A	+	+	
79		Bagridae	*Pseudobagrus*	*P. pratti*	N	B	C	A	+	+	
80				*P. brevicaudatus*	N	B	C	A	+	+	
81			*Tachysurus*	*T. fulvidraco*	N	L	O	A	+	+	+
82				*T. nitidus*	N	L	O	A	+	+	+
83			*Leiocassis*	*L. crassilabris*	N	B	C	A	+	+	+
84			*Hemibagrus*	*H. macropterus*	R	B	C	A	+	+	+
85		Amblycipitidae	*Liobagrus*	*L. marginatus*	R	B	C	A	+	+	
86				*L. nigricauda*	R	B	C	A	+		
87		Sisoridae	*Glyptothorax*	*G. sinensis*	R	B	O	A	+		
88	Cyprinodontiformes	Poeciliidae	*Gambusia*	*G. affinis**	N	B	C	S	+	+	+
89	Synbgranchiformes	Synbranchidae	*Monopterus*	*M. albus*	N	B	C	F		+	
90	Perciformes	Channidae	*Channa*	*C. argus*	N	B	C	P	+	+	+
91		Gobiidae	*Rhinogobius*	*R. cliffordpopei*	G	B	C	D	+	+	+
92			*Mugilogobius*	*M. myxodermus*	N	B	C	D		+	+
93		Belontiidae	*Macropodus*	*M. opercularis*	N	L	O	P		+	
94		Cichlidae	*Coptodon*	*C. zillii**	G	B	O	S	+	+	+
95			*Oreochromis*	*O. niloticus**	G	B	O	S			+
96		Serranidae	*Siniperca*	*S. chuatsi*	N	L	C	P	+	+	
97				*S. scherzeri*	G	L	C	P	+	+	+
98		Centrarchidae	*Micropterus*	*M. salmoides**	N	L	O	A		+	
99	Salmoniformes	Salangidae	*Neosalanx*	*N. taihuensis**	N	U	C	D		+	
Total	7	20	74	99					86	81	58

Note: *: alien fish, ●: The endemic fish of the upper Yangtze River, ★: Nationally protected fish, N: semi-lentic water; R: flowing water; G: eurytopicity; H: herbivory; O: omnivory; C: carnivority; U: pelagic fish; L: benthopelagic fish; B: demersal fish; A: adhesive eggs; D: demersal eggs; F: drifting eggs; P: floating eggs; S: special modality

Supplementary Table S3 The sequence count per sample before and after filtration

	before	after		before	after
ZL1	614841	304413	JX1	566044	204771

ZL2	693362	406610	JX2	607276	213506
ZL3	693133	276866	JX3	564768	263661
JT1	559154	142833	MH1	684831	120113
JT2	640362	138015	MH2	657907	222132
JT3	640361	240441	MH3	574919	154696
BMG1	665353	422397	FY1	677697	234841
BMG2	668712	428406	FY2	665550	207597
BMG3	582004	312962	FY3	664131	210056
SSP1	593873	314818	XLM1	631129	287823
SSP2	696401	434806	XLM2	674467	267593
SSP3	678968	430799	XLM3	555229	228991
TZK1	600473	206404	QJ1	642271	275729
TZK2	653398	232026	QJ2	555207	180613
TZK3	567739	195832	QJ3	567974	189462
CX1	676570	161719	DXG1	583104	286684
CX2	680339	187078	DXG2	573771	252111
CX3	628821	158392	DXG3	558760	287090
SX1	650759	214350	TZH1	617280	213139
SX2	660716	220771	TZH2	614411	222022
SX3	651272	237064	TZH3	650301	239858
JYT1	651609	370141	LZ1	583353	208971
JYT2	643735	402136	LZ2	593573	263026
JYT3	603889	343730	LZ3	684814	288566
HYZ1	640638	447963	CJ1	601392	180010
HYZ2	627172	392726	CJ2	584836	140284
HYZ3	598338	369231	CJ3	595469	162772
XZ1	557567	367966	RJD1	551818	190821
XZ2	691830	398253	RJD2	628404	189705
XZ3	652287	334852	RJD3	681714	118580

Supplementary Table S4 The eDNA technology detected fish species and their taxonomic status, as well as the sequence numbers of these fish detected in each sampling area

Serial number	Order	Family	Genus	Species	ZL	JT	BMG	SSP	TZK	CX	SX	JYT	HYZ	XZ	JX	MH	FY	XLM	QJ	DXG	TZH	LZ	CJ	RJD
1	Acipenseriformes	Acipenseridae	*Acipenser*	*A.* sp.	0	0	2	2301	0	0	0	0	0	0	0	0	543	0	0	0	0	0	0	0
2	Cypriniformes	Botiidae	*Parabotia*	*P. fasciatus*	0	0	4513	6471	1965	4	0	0	0	0	0	0	1834	1	0	1	0	0	0	0
3			*Sinibotia*	*S. superciliaris*	0	0	46856	32317	0	0	0	0	0	0	0	0	0	0	2	1	0	0	0	0
4				*S. reevesae*●	0	0	1760	412	0	0	0	0	0	0	0	0	0	0	0	0	0	0	0	0
5			*Leptobotia*	*L. elongata*★●	0	0	3352	932	1	0	0	0	0	0	0	0	1	0	0	0	0	0	0	0
6		Cobitidae	*Triplophysa*	*T. orientalis*	18648	6062	0	0	0	0	0	0	0	0	0	0	0	0	0	0	0	0	0	0
7				*T. bleekeri*●	1065	6	0	0	0	0	0	0	0	0	0	0	0	0	0	0	0	0	0	0
8				*T. stenura*	1	0	0	0	0	0	0	0	0	0	0	0	0	0	0	0	0	0	0	0
9			*Homatula*	*H. variegata*	112	55	4	4	0	0	0	0	0	0	0	0	0	0	0	0	0	0	0	0
10				*H. potanini*●	33881	9785	1352	851	0	0	0	0	0	0	0	0	0	0	0	0	0	0	0	0
11			*Misgurnus*	*M. anguillicaudatus*	2031	999	0	0	0	0	282	1	0	0	0	0	0	0	1039	0	1	1779	1	3065
12			*Paramisgurnus*	*P. dabryanus*	5	3	1	0	0	1	6	4445	18	13201	0	0	0	0	1764	2	2675	0	0	1
13		Cyprinidae	*Zacco*	*Z. platypus*	68605	27324	3157	729	15	1805	39	23	17	15	14	16	44	909	19	16	12	43	14	5452
14			*Opsariichthys*	*O. bidens*	19358	17092	2988	1310	1	0	0	1	6160	1229	3604	22416	35299	30084	7673	4020	16400	9743	833	139
15			*Ctenopharyngodon*	*C. idella*	15553	1630	3996	2876	6992	5197	30646	40065	10473	18653	19189	2985	43566	10205	18239	25518	11697	2908	10070	8529
16			*Mylopharyngodon*	*M. piceus*	7	1	2	9	1	3	0	1	859	209	0	0	1	546	1690	1	0	0	0	2
17			*Squaliobarbus*	*S. curriculus*	11	2	36577	45153	2321	10	11	39	7827	6221	5183	8	27	7	17	1708	18283	19549	35095	3227

18	*Rhynchocypris*	*R. lagowskii*	44732	4512	877	1927	3211	4	0	0	0	0	0	0	0	0	0	0	0	0	0	0
19	*Tinca*	*T. tinca**	3	0	0	1	0	0	0	0	0	0	0	0	0	0	0	0	0	0	0	0
20	*Elopichthys*	*E. bambusa*	0	0	0	0	0	0	0	0	952	3	0	0	0	0	0	0	0	0	605	194
21	*Ochetobius*	*O. elongatus*	0	0	15	26	0	0	0	0	2	4	1	0	2036	3	0	2	9	23	28	0
22	*Hemiculterella*	*H. sauvagei*•	16	2290	6303	2321	0	1	0	2	0	0	0	0	3805	344	1	0	0	0	0	1
23	*Hemiculter*	*H. leucisculus*	10	3	10	75	1763	151	1158	4475	232176	869	3448	4144	576	1783	3498	1129	8900	2043	3866	2476
24		*H. tchangi*•	1082	7	690	17451	14618	38482	30499	72824	10763	14532	29021	6813	8426	114342	8949	7574	17931	15100	10461	7699
25	*Pseudohemiculter*	*P. dispar**	14	1	6	0	65	39	1	50	0	0	52	0	0	24	0	0	30	1	3	658
26	*Ancherythroculter*	*A. wangi*•	1780	5985	3504	11376	9198	13445	3214	5726	1038	1290	3394	7975	6013	937	5328	2466	5813	14	1473	3154
27	*Pseudolaubuca*	*P. sinensis*	0	0	0	10	0	0	643	2	6	2705	2398	2	832	725	526	5	6314	27	6	2121
28		*P. engraulis*	1	3	2862	4874	0	591	2229	979	0	0	853	0	0	0	0	0	0	0	0	0
29	*Chanodichthys*	*C. erythropterus*	2	1308	0	1	5	1551	3	704	0	0	0	0	3572	441	0	0	0	0	0	0
30		*C. alburnus*	1	1	1159	3995	8083	5024	3138	11	460	2445	0	0	4	0	903	2	3116	0	845	2146
31		*C. oxycephaloides*	0	5	21	4468	60	3122	566	3499	1	7	1	0	0	1	2	2	20	0	3	17
32		*C. dabryi*	0	0	1013	12	1	15	1	0	992	4	0	0	0	0	1	0	704	1	0	1721
33	*Megalobrama*	*M. amblycephala**	24	303	7	1	3261	37	0	41	1	0	29	1	3827	19	0	0	2746	6	0	2367
34		*M. terminalis**	1	140	0	0	6	1	0	1	0	0	0	0	0	0	0	0	0	0	0	1355
35	*Xenocypris*	*X. davidi*	663	3075	7202	11438	108	319	12	98	146	1176	1836	1	291	1173	325	260	1100	4935	1255	6
36		*X. yunnanensis*	35	190	165585	148591	4086	10928	553	3911	7260	25545	46510	13	11405	37136	11965	7554	40016	152050	31458	17
37	*Plagiognathops*	*P. microlepis*	1	0	0	5	2	9	19	17	12	3	10	1	67	89	1	2	2	112	3	2
38	*Distoechodon*	*D. tumirostris*	4528	873	2955	8031	2012	6	17	0	627	5	0	3905	1	0	1	1119	4	3	0	1
39	*Parabramis*	*P. pekinensis*	8	722	71	335	2301	4573	6	10	26	3891	1465	10	25	680	22	14	2759	14	7	1288

40	*Pseudobrama*	*P. simoni*	3	1	9	2455	1989	5106	15482	11627	4994	2924	4396	3	3600	4189	18935	23799	7654	5165	14029	1489
41	*Acheilognathus*	*A. macropterus*	3	562	0	0	1	2	17212	70292	9504	6596	228	12859	11392	18818	15173	4626	7641	5	1	0
42		*A. omeiensis*●	0	0	0	0	0	0	0	0	2	536	0	0	0	0	0	0	0	0	0	0
43	*Acanthorhodeus*	*A. chankaensis*	1	0	1	0	0	0	2927	7811	4133	1503	1	0	0	0	979	350	0	0	0	1
44	*Rhodeus*	*R. sinensis*	11	12	2	2	1	1	10	7	694	8	1683	4	4	1354	21	3480	1025	4	1	2077
45		*R. ocellatus*	529	1	1	3	0	1	911	112	2009	1994	1537	1139	9	1278	1069	133540	3992	1862	1470	631
46	*Spinibarbus*	*S. caldwelli*	0	0	0	0	0	0	0	0	0	0	0	0	0	806	0	0	0	0	0	0
47		*S. sinensis*●	8	1283	18084	8573	4	2252	0	0	14	4074	1	0	0	3	1	1	943	977	1	2195
48	*Acrossocheilus*	*A. monticola*●	6	5	6	17	0	0	0	0	0	0	0	0	0	0	0	0	0	0	0	0
49	*Onychostoma*	*O. macrolepis*★	33116	24243	8918	3804	0	0	0	0	0	0	0	0	0	0	0	1	0	0	0	0
50	*Hemibarbus*	*H. maculatus*	36	33	4	7	0	15	34	345	31	7	75	0	0	2	139	0	0	0	2	0
51		*H. labeo*	297	162	1859	4550	8	4093	9762	9181	615	5839	1259	1	1	4	2708	6	0	0	1873	0
52	*Belligobio*	*B. nummifer*	0	0	0	0	0	1	2	1	0	0	0	0	0	0	0	0	0	0	0	0
53	*Pseudorasbora*	*P. parva**	6093	779	0	0	285	1111	364	0	1117	1	0	4665	1432	825	1214	0	0	0	1662	397
54	*Sarcocheilichthys*	*S. sinensis*	0	0	0	0	0	0	2	3365	648	2	642	1	0	0	0	0	0	0	0	0
55		*S. nigripinnis*	4	2207	0	0	0	0	0	0	2	0	0	0	0	0	0	0	0	0	0	0
56	*Gnathopogon*	*G. herzensteini*●	6410	230	669	31	0	0	0	0	0	0	0	0	0	0	0	0	0	0	0	1
57	*Coreius*	*C. heterodon*	0	0	0	0	0	0	0	0	0	0	0	0	0	0	0	0	0	0	0	288
58		*C. guichenoti*★●	0	1	3	2	0	1	0	0	0	0	0	0	896	1	1	2	3	844	0	0
59	*Squalidus*	*S. argentatus*	4	1	2742	3620	2707	1323	329	7774	12730	624	37	0	0	17	4024	1138	1	1055	1	1
60		*S. wolterstorffi*	253	39	7	12	0	0	0	0	1	1	0	0	0	0	0	0	0	0	3	0
61	*Rhinogobio*	*R. typus*	54	503	21792	10331	0	37	0	0	7	0	27	0	0	0	1327	19	2898	349	1902	5

62				*R. cylindricus*●	4	1158	361	222	3	1547	0	0	1112	2	1875	0	0	0	2179	1454	89	2557	62	2236
63			*Abbottina*	*A. rivularis*	20516	4092	1	0	0	0	10326	698	9012	14	1	1129	0	0	2829	7457	0	0	0	0
64			*Microphysogobio*	*M. kiatingensis*	780	412	367	2568	1	1	3	3	13	2	2	1	2	2	12469	2552	0	3	1	3
65			*Saurogobio*	*S. dabryi*	0	0	272	2	2525	9557	4393	5064	2381	3619	4838	1	396	2902	19345	1037	2	1845	2648	0
66			*Gobiobotia*	*G. filifer*	0	0	976	885	0	0	0	0	0	0	0	0	0	0	0	0	0	0	0	0
67			*Schizothorax*	*S. davidi*★●	36	1852	1	1	0	0	0	0	0	0	0	0	0	0	0	0	0	0	0	0
68			*Carassius*	*C. auratus*	229	780	1107	341	4873	1122	2796	473	4895	1452	1919	302	306	4922	2293	814	217	69	59	2503
69			*Cyprinus*	*C. carpio*	15669	18071	6960	12345	18950	29762	62501	44316	27229	168691	51198	31333	23825	7818	26705	10066	12940	5981	13437	34359
70			*Procypris*	*P. rabaudi*★●	0	0	0	0	0	0	0	0	0	0	809	1	0	0	0	0	0	0	0	0
71			*Hypophthalmichthys*	*H. molitrix*	1576	2176	597	1553	6401	3248	853	7829	1800	19	2938	131	307	1134	8679	1140	33	94	6314	10459
72			*Aristichthys*	*A. nobilis*	5793	4704	3508	9429	94877	9063	5399	19645	1814	137	5661	18757	10678	6811	14257	4005	1560	3098	4541	23609
73		Balitoridae	*Jinshaia*	*J. abbreviata*●	19	11	450	25	0	0	0	0	0	0	0	0	0	0	0	0	0	0	0	0
74			*Sinogastromyzon*	*S. szechuanensis*●	5455	3311	7079	3089	0	0	0	0	0	0	0	0	0	0	0	0	0	0	0	0
75				*S. sichangensis*●	6	4	7	2	0	0	0	0	0	0	0	0	0	0	0	0	0	0	0	0
76	Siluriformes	Siluridae	*Silurus*	*S. asotus*	4	575	1950	24	5563	373	2985	663	45	33578	2021	2	0	446	0	0	2	1087	0	0
77				*S. meridionalis*	7	7	2	1	6	11	9	1	5	20	1640	0	0	0	0	0	1	3	2	0
78		Ictaluridae	*Ictalurus*	*I. punctatus**	0	222	0	0	0	0	1347	1	0	0	0	0	0	0	0	0	0	0	0	0
79		Bagridae	*Pseudobagrus*	*P. pratti*	15065	8754	1915	1136	3334	3	1	1	0	2	0	1	1	1	0	1	0	0	0	0
80				*P. brevicaudatus*	2	749	0	3	372	901	249	5	6	3	0	3	0	0	0	2	4	0	0	0
81			*Tachysurus*	*T. fulvidraco*	2057	1801	647	1	2774	910	0	0	2	1305	3336	3	5184	1087	2514	7	1	0	3	8492
82				*T. nitidus*	4	10	0	12	4	718	1	0	1728	1153	3	2570	0	0	1	1	0	0	891	79
83			*Leiocassis*	*L. crassilabris*	1	3	3355	6679	4522	775	1104	4653	3393	1331	0	8	0	3	4	1361	3341	2	4	5

84			*Hemibagrus*	*H. macropterus*	2	3	1653	5118	1	0	1	222	660	2760	1926	4864	1035	785	0	1	9401	1	1	2520
85		Amblycipitidae	*Liobagrus*	*L. marginatus*	0	0	1220	1455	1	700	0	0	0	0	0	0	0	0	0	0	0	0	0	0
86				*L. nigricauda*	0	0	3	3	0	0	0	0	0	0	0	0	0	0	0	0	0	0	0	0
87		Sisoridae	*Glyptothorax*	*G. sinensis*	418	3188	2424	1575	0	0	0	0	0	0	0	0	0	0	0	0	0	0	0	0
88	Cyprinodontiformes	Poeciliidae	*Gambusia*	*G. affinis**	1	0	0	0	0	0	649	1	272	1	0	0	0	0	1710	2791	0	0	0	1280
89	Synbgranchiformes	Synbranchidae	*Monopterus*	*M. albus*	0	0	0	0	0	0	0	0	1	1211	0	0	0	0	0	0	0	0	0	0
90	Perciformes	Channidae	*Channa*	*C. argus*	1	2	0	0	0	0	626	2008	1511	1	377	0	3	1170	3344	798	6	9193	0	392
91		Gobiidae	*Rhinogobius*	*R. cliffordpopei*	1809	4953	919	4580	50	9376	5679	31033	23391	27525	18861	12074	19149	4313	4581	7977	30090	5044	10797	4619
92			*Mugilogobius*	*M. myxodermus*	0	0	0	0	0	0	1	0	4203	5028	2935	15165	12730	3210	2605	9656	4545	4085	2238	1443
93		Belontiidae	*Macropodus*	*M. opercularis*	0	0	0	0	0	0	0	0	0	0	0	0	0	0	1	786	0	0	0	0
94		Cichlidae	*Coptodon*	*C. zillii**	759	1	1131	3	0	0	0	0	0	0	0	8390	4263	3	2062	2	1	1758	2984	12399
95			*Oreochromis*	*O. niloticus**	0	0	0	0	0	0	0	0	0	0	0	0	0	0	0	0	0	0	1	9188
96		Serranidae	*Siniperca*	*S. chuatsi*	1	7	0	1	0	520	363	7695	3421	2341	0	3795	0	0	387	3666	0	0	0	0
97				*S. scherzeri*	2	4393	1	646	1	3	3356	170	30	617	0	81	0	0	1044	1269	0	0	0	2
98		Centrarchidae	*Micropterus*	*M. salmoides**	0	0	0	0	2021	3	490	0	0	0	0	0	0	0	0	0	0	0	0	0
99	Salmoniformes	Salangidae	*Neosalanx*	*N. taihuensis**	0	0	0	0	3	1147	809	1	0	0	0	0	0	0	605	0	0	0	0	0
	Number of fish species in each river section				71	69	68	71	50	56	54	53	58	56	45	41	42	46	53	53	45	41	45	51

Note: *: alien fish, ●: The endemic fish of the upper Yangtze River, ★: Nationally protected fish.

Supplementary Table S5 The dominant sequence species in each river section. Additionally, count the total number of dominant fish species in each river sections and calculate the proportion of fish preferring flowing water and small-bodied fish among them

River sections	Sequence dominant species (McNaughton dominance index)	Total	Percentage of fish preferring flowing water	Percentage of small-bodied fish
ZL	*Z. platypus*△√ (0.2083), *R. lagowskii*△ (0.1358), *H. potanini*△√ (0.1029), *O. macrolepis* (0.1005), *A. rivularis*△ (0.0623), *O. bidens*△√ (0.0588), *T. orientalis*△√ (0.0566), *C. carpio* (0.0476), *C. idella* (0.0472), *P. pratti*△ (0.0458)	10	50%	70%
JT	*Z. platypus*△√ (0.1573), *O. macrolepis*√ (0.1395), *C. carpio* (0.1040), *O. bidens*△√ (0.0985), *H. potanini*△√ (0.0563), *P. pratti*△ (0.0504), *T. orientalis*△√ (0.0349), *A. wangi*√ (0.0344), *R. cliffordpopei*△ (0.0285), *A. nobilis* (0.0271), *R. lagowskii*△ (0.0260), *S. scherzeri* (0.0253), *A. rivularis*△ (0.0235)	13	46.15%	61.54%
BMG	*X. yunnanensis*△ (0.4269), *S. superciliaris*△√ (0.1208), *S. curriculus* (0.0943), *R. typus*△√ (0.0562), *S. sinensis*√ (0.0466), *O. macrolepis*√ (0.0230)	6	66.67%	50%
SSP	*X. yunnanensis*△ (0.3776), *S. curriculus* (0.1147), *S. superciliaris*△√ (0.0821), *H. tchangi*△ (0.0444), *C. carpio* (0.0314), *X. davidi* (0.0291), *A. wangi*√ (0.0289), *R. typus*△√ (0.0263), *A. nobilis* (0.0240), *S. sinensis*√ (0.0218), *D. tumirostris* (0.0204)	11	36.36%	36.36%
TZK	*A. nobilis* (0.4488), *C. carpio* (0.0896), *H. tchangi*△ (0.0692), *A. wangi*√ (0.0435), *C. alburnus* (0.0382), *C. idella* (0.0331), *H. molitrix* (0.0303), *S. asotus* (0.0263), *C. auratus*△ (0.0230), *L. crassilabris*△ (0.0214)	10	10%	30%
CX	*H. tchangi*△ (0.2277), *C. carpio* (0.1761), *A. wangi*√ (0.0795), *X. yunnanensis*△ (0.0647), *S. dabryi*△ (0.0565), *R. cliffordpopei*△ (0.0555), *A. nobilis* (0.0536), *C. idella* (0.0308), *P. simoni*△ (0.0302), *C. alburnus* (0.0297), *P. pekinensis* (0.0270), *H. labeo* (0.0242)	12	8.33%	41.67%
SX	*C. carpio* (0.2789), *C. idella* (0.1368), *H. tchangi*△ (0.1361), *A. macropterus*△ (0.0768), *P. simoni*△ (0.0691), *A. rivularis*△ (0.0461), *H. labeo* (0.0436), *R. cliffordpopei*△ (0.0254), *A. nobilis* (0.0241)	9	0	55.56%
JYT	*H. tchangi*△ (0.1958), *A. macropterus*△ (0.1890), *C. carpio* (0.1191), *C. idella* (0.1077), *R. cliffordpopei*△ (0.0834), *A. nobilis* (0.0528),	12	8.33%	50%

	P. simoni△ (0.0313), *H. labeo* (0.0247), *H. molitrix* (0.0211), *A. chankaensis*△ (0.0210), *S. argentatus*△√ (0.0209), *S. chuatsi* (0.0207)			
HYZ	*H. leucisculus*△ (0.5757), *C. carpio* (0.0675), *R. cliffordpopei*△ (0.0580), *S. argentatus*△√ (0.0316), *H. tchangi*△ (0.0267), *C. idella* (0.0260), *A. macropterus*△ (0.0236), *A. rivularis*△ (0.0223)	8	12.5%	75%
XZ	*C. carpio* (0.4596), *S. asotus* (0.0915), *R. cliffordpopei*△ (0.0750), *X. yunnanensis*△ (0.0696), *C. idella* (0.0508), *H. tchangi*△ (0.0396), *P. dabryanus*△ (0.0360)	7	0	57.14%
JX	*C. carpio* (0.2253), *X. yunnanensis*△ (0.2046), *H. tchangi*△ (0.1277), *C. idella* (0.0844), *R. cliffordpopei*△ (0.0830), *A. nobilis* (0.0249), *S. curriculus* (0.0228), *S. dabryi*△ (0.0213)	8	0	50%
MH	*C. carpio* (0.1891), *O. bidens*△√ (0.1355), *A. nobilis* (0.1132), *M. myxodermus*△ (0.0916), *A. macropterus*△ (0.0776), *R. cliffordpopei*△ (0.0729), *A. wangi*√ (0.0482), *H. tchangi*△ (0.0412), *C. zillii* (0.0337), *H. leucisculus*△ (0.0250)	10	20%	60%
FY	*C. idella* (0.2003), *O. bidens*△√ (0.1624), *C. carpio* (0.1096), *R. cliffordpopei*△ (0.0881), *M. myxodermus*△ (0.0585), *X. yunnanensis*△ (0.0525), *A. macropterus*△ (0.0524), *A. nobilis* (0.0492), *H. tchangi*△ (0.0388), *A. wangi*√ (0.0277), *T. fulvidraco*△ (0.0238)	11	18.18%	63.64%
XLM	*H. tchangi*△ (0.4373), *X. yunnanensis*△ (0.1421), *O. bidens*△√ (0.1152), *A. macropterus*△ (0.0720), *C. idella* (0.0390), *C. carpio* (0.0299), *A. nobilis* (0.0261)	7	14.29%	57.14%
QJ	*C. carpio* (0.1241), *S. dabryi*△ (0.0899), *P. simoni*△ (0.0880), *C. idella* (0.0847), *A. macropterus*△ (0.0705), *A. nobilis* (0.0662), *M. kiatingensis*△√ (0.0579), *X. yunnanensis*△ (0.0556), *H. tchangi*△ (0.0416), *H. molitrix* (0.0403), *O. bidens*△√ (0.0357), *A. wangi*√ (0.0248), *R. cliffordpopei*△ (0.0213)	13	23.08%	61.54%
DXG	*R. ocellatus*△ (0.4851), *C. idella* (0.0927), *P. simoni*△ (0.0865), *C. carpio* (0.0366), *M. myxodermus*△ (0.0351), *R. cliffordpopei*△ (0.0290), *H. tchangi*△ (0.0276), *X. yunnanensis*△ (0.0275), *A. rivularis*△ (0.0271)	9	0	77.78%
TZH	*X. yunnanensis*△ (0.1779), *R. cliffordpopei*△ (0.1338), *S. curriculus* (0.0813), *H. tchangi*△ (0.0797), *O. bidens*△√ (0.0730), *C. carpio* (0.0575), *C. idella* (0.0520), *H. leucisculus*△ (0.0396), *P. simoni*△ (0.0340), *A. macropterus*△ (0.0340), *P. sinensis*△ (0.0281), *H. macropterus*√ (0.0278), *A. wangi*√ (0.0258)	13	23.08%	61.54%
LZ	*X. yunnanensis*△ (0.5998), *S. curriculus* (0.0771), *H. tchangi*△ (0.0596), *O. bidens*△√ (0.0385), *C. argus* (0.0363), *C. carpio* (0.0236), *P. simoni*△ (0.0204)	7	14.29%	57.14%

CJ	*S. curriculus* (0.2180), *X. yunnanensis*△ (0.1954), *P. simoni*△ (0.0871), *C. carpio* (0.0835), *R. cliffordpopei*△ (0.0671), *H. tchangi*△ (0.0650), *C. idella* (0.0626), *H. molitrix* (0.0392), *A. nobilis* (0.0282), *H. leucisculus*△ (0.0240)	10	0	50%
RJD	*C. carpio* (0.2065), *A. nobilis* (0.1419), *C. zillii* (0.0745), *H. molitrix* (0.0629), *O. niloticus* (0.0552), *C. idella* (0.0513), *T. fulvidraco*△ (0.0510), *H. tchangi*△ (0.0463), *Z. platypus*△√ (0.0328), *R. cliffordpopei*△ (0.0278)	10	10%	40%
UR	*X. yunnanensis*△ (0.2448), *Z. platypus*△√ (0.0777), *S. curriculus* (0.0636), *O. macrolepis*√ (0.0546), *C. carpio* (0.0413), *R. lagowskii*△ (0.0405), *H. potanini*△√ (0.0357), *O. bidens*△√ (0.0318), *S. superciliaris*△√ (0.0308), *R. typus*△√ (0.0254), *S. sinensis*√ (0.0218), *P. pratti*△ (0.0209)	12	58.33%	66.67%
MR	*C. carpio* (0.1453), *H. tchangi*△ (0.1087), *X. yunnanensis*△ (0.1001), *H. leucisculus*△ (0.0742), *C. idella* (0.0687), *R. cliffordpopei*△ (0.0555), *A. nobilis* (0.0546), *A. macropterus*△ (0.0463), *R. ocellatus*△ (0.0377), *O. bidens*△√ (0.0354), *P. simoni*△ (0.0292)	11	9.09%	72.73%
LR	*C. carpio* (0.1460), *S. curriculus* (0.1171), *X. yunnanensis*△ (0.0962), *A. nobilis* (0.0860), *C. idella* (0.0568), *H. tchangi*△ (0.0555), *H. molitrix* (0.0512), *P. simoni*△ (0.0474), *R. cliffordpopei*△ (0.0471), *C. zillii* (0.0470), *T. fulvidraco*△ (0.0259)	11	0	45.45%

Note: △ for small-bodied fish, √ for fish preferring flowing water.
